# Care to Quit: a stepped wedge cluster randomised controlled trial to implement best practice smoking cessation care in cancer centres

**DOI:** 10.1186/s13012-021-01092-5

**Published:** 2021-03-04

**Authors:** Christine L. Paul, Graham Warren, Shalini Vinod, Bettina Meiser, Emily Stone, Daniel Barker, Kate White, James McLennan, Fiona Day, Kristen McCarter, Melissa McEnallay, Jordan Tait, Karen Canfell, Marianne Weber, Catherine Segan

**Affiliations:** 1 University of Newcastle Priority Research Centre for Cancer Research, Innovation and Translation, Callaghan, Australia; 2 University of Newcastle School of Medicine and Public Health, Callaghan, NSW Australia; 3 Level 4 West, Hunter Medical Research Institute, John Hunter Hospital, New Lambton Heights, Newcastle, NSW Australia; 4 Hunter Cancer Research Alliance, John Hunter Hospital, New Lambton Heights, Newcastle, NSW Australia; 5 Department of Radiation Oncology, Department of Cell and Molecular Pharmacology, Medical University of South Carolina, Charleston, SC USA; 6 Cancer Therapy Centre, Liverpool Hospital, Liverpool, NSW Australia; 7 South Western Sydney Clinical School and Ingham Institute for Applied Medical Research, Liverpool, NSW Australia; 8 Prince of Wales Clinical School, University of New South Wales, Kensington, NSW 2052 Australia; 9 St Vincent’s Hospital Sydney, Kinghorn Cancer Centre, University of NSW, Kensington, Australia; 10 Faculty of Medicine and Health, University of Sydney, CNRU Sydney Local Health District, Sydney, Australia; 11 St Vincent’s Hospital Sydney, Darlinghurst, NSW Australia; 12 Calvary Mater Newcastle, Hunter Region Mail Centre, Waratah, NSW Australia; 13 Cancer Research Division, Cancer Council NSW, Woolloomooloo, NSW Australia; 14 Prince of Wales Clinical School, University of New South Wales, Sydney, NSW Australia; 15 Sydney School of Public Health, University of Sydney, Sydney, NSW Australia; 16 Cancer Council Victoria, Melbourne, VIC Australia; 17 Centre for Health Policy, Melbourne School of Population and Global Health, The University of Melbourne, Melbourne, VIC Australia

**Keywords:** Smoking, Smoking cessation, Cancer, Implementation, Quitline

## Abstract

**Background:**

Cigarette smoking in people with cancer is associated with negative treatment-related outcomes including increased treatment toxicity and complications, medication side effects, decreased performance status and morbidity. Evidence-based smoking cessation care is not routinely provided to patients with cancer. The purpose of this study is to determine the effectiveness of a smoking cessation implementation intervention on abstinence from smoking in people diagnosed with cancer.

**Methods:**

A stepped wedge cluster randomised design will be used. All sites begin in the control condition providing treatment as usual. In a randomly generated order, sites will move to the intervention condition. Based on the Theoretical Domains Framework, implementation of Care to Quit will include (i) building the capability and motivation of a critical mass of key clinical staff and identifying champions; and (ii) identifying and implementing cessation care models/pathways. Two thousand one hundred sixty patients with cancer (diagnosed in the prior six months), aged 18+, who report recent combustible tobacco use (past 90 days or in the 30 days prior to cancer diagnosis) and are accessing anti-cancer therapy, will be recruited at nine sites. Assessments will be conducted at baseline and 7-month follow-up. The primary outcome will be 6-month abstinence from smoking. Secondary outcomes include biochemical verification of abstinence from smoking, duration of quit attempts, tobacco consumption, nicotine dependence, provision and receipt of smoking cessation care, mental health and quality of life and cost effectiveness of the intervention.

**Discussion:**

This study will implement best practice smoking cessation care in cancer centres and has the potential for wide dissemination.

**Trial registration:**

The trial is registered with ANZCTR (www.anzctr.org.au): ACTRN (ACTRN12621000154808) prior to the accrual of the first participant and will be updated regularly as per registry guidelines.

**Supplementary Information:**

The online version contains supplementary material available at 10.1186/s13012-021-01092-5.

Contributions to the literature

- There has been little research conducted into the most effective systematic implementation of smoking cessation care in cancer centres.
- The implementation intervention to be employed in this trial has been informed by the Theoretical Domains Framework.
- The results of this trial will inform models of implementation of best practice smoking cessation care in cancer settings.

## Background

Cigarette smoking in people with cancer is associated with a host of deleterious treatment-related outcomes including increased treatment toxicity and complications [1–5], medication side effects [6], hospitalisation [7–9], decreased performance status [10] and morbidity [11, 12]. These adverse health effects lead to increased symptom burden and toxicity such as a 20% greater chance of radiation pneumonitis [13], double the rate of laryngeal complications [5], and greater mucositis [14]. Continued smoking (compared to quitting at diagnosis) doubles risk of death and halves median survival time [13]. Associations between continued smoking and poorer outcomes have been identified for various cancer types, with abstinence from smoking being the strongest predictor of survival in cancer patients, other than tumour site and stage at diagnosis [15]. Despite a strong desire to quit [16], most people with cancer who smoke do not achieve abstinence [15]. Consequently, evidence-based smoking cessation support may improve cancer outcomes.

In response to the US Surgeon General’s 2014 report [17], the US National Comprehensive Cancer Network produced the Clinical Practice Guidelines in Oncology for Smoking Cessation [18]. It is widely recognised that evidence-based quality cancer care includes addressing tobacco use [19, 20], and there is a strong rationale for all cancer patients to be screened for their smoking status, advised of the health benefits of cessation and provided with help to quit [21]. Additionally, high smoking relapse rates and the mis-reporting of smoking status in the oncology setting [22] indicate the need to provide such support to those who report recently quitting smoking as well as those who report being current smokers. A meta-analysis of the eight smoking cessation trials conducted with people with cancer indicated that trials using combination therapy (i.e. pharmacotherapy combined with behavioural therapies such as telephone support) were more effective than those without pharmacotherapy [23].

An Australian survey showed that over 85% of people with cancer who smoke agreed that health professionals should provide assistance to help them quit smoking (prior unpublished work Sherwood, Tzelepis, Day and Paul et al. unpublished) More than 80% of US and Australian oncology staff agree that smoking cessation support should be part of cancer care [24–26]. However, large surveys of mainly US-based clinicians demonstrated that while approximately 90% of oncologists ask about tobacco use and 80% advise patients to stop smoking, less than 40% discuss medications or assist patients in cessation [15, 27]. A national Australian survey of oncologists (*n* = 685) found that while 94% agree that smoking impacts treatment outcomes and 95% ask about smoking status most of the time, few offer evidence-based cessation support strategies [26]. Only 16% commonly discuss cessation medications and 18% make referrals to cessation support [26].

The dominant barriers to delivering cessation care to people with cancer are lack of time, expertise and resources [28]. There has been little research conducted into addressing these barriers with effective systematic implementation of smoking cessation care in cancer centres [21]. In order for the potential benefits of smoking cessation for people with cancer to be realised, there is a need to identify models of implementation of best practice smoking cessation care in cancer settings. Informed by the Theoretical Domains Framework [29], the Care to Quit project will trial a model that is designed to be implemented in busy oncology settings including engaging and equipping key health professionals to make smoking cessation care a priority, and to deliver pivotal brief advice and referral (requiring minimal time at each patient encounter) as well as engaging the wider clinical team through individualised teaching on how to make smoking cessation care routine; building and promoting the capability of Quitline to deliver supportive cessation care to people with cancer; and identifying and implementing workflows and patient pathways which continuously link patients to the most effective forms of cessation care (multi-session behavioural therapy and pharmacotherapy).

Care to Quit is a stepped wedge cluster randomised trial of an implementation intervention of best practice cessation care versus treatment as usual in nine Australian hospitals that provide care to patients with cancer. The specific aims of the Care to Quit project are to compare the effect of a smoking cessation care implementation intervention in cancer centres on (1) smoking cessation outcome measures; (2) provision of smoking cessation care and mental health and quality of life; and (3) the cost effectiveness of the intervention. We will also assess process measures such as Quitline referrals, staff attitudes, acceptability and system perspectives.

### Primary hypotheses

1a) The proportion of smokers and recent quitters receiving anti-cancer therapy who achieve self-reported 6-month prolonged abstinence from smoking will be higher post-intervention compared to pre-intervention (14% vs. 7%).

1b) Biochemically verified 7-day point-prevalence abstinence from smoking at 7 months post-recruitment will be higher after the intervention phase than at the baseline phase (25% vs. 15%).

### Secondary hypotheses

2) The proportion of smokers and recent quitters who are provided the following will be higher after the intervention phase, compared to the baseline phase:

- Advice from members of their multidisciplinary team about the cancer-specific benefits of stopping smoking or staying quit (80% v 60%)
- Proactive Quitline referral, i.e. arrange for Quitline to call patient (45% vs. 10%)
- Prescription or provision of pharmacotherapy (35% vs. 15%)

3) The implementation intervention will be cost effective compared to usual care.

## Methods/design

### Study setting

The study is being conducted in nine Australian hospitals (across New South Wales and Victoria) that provide care to patients with cancer.

### Study design

**Fig. 1:**
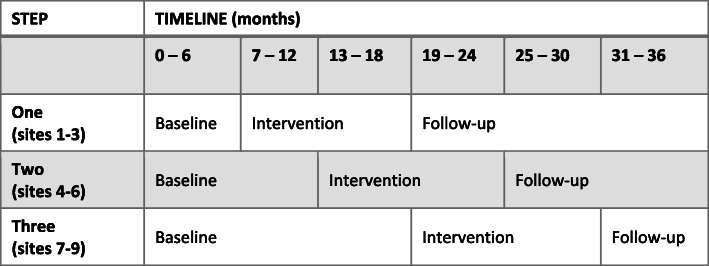
Stepped-wedge model

Care to Quit is a stepped wedge cluster randomised trial of an implementation intervention of best practice smoking cessation care versus treatment as usual in nine Australian hospitals that provide care to patients with cancer. All sites (hospitals) begin as part of the control condition and are block randomised. At each intervention ‘step’, three sites will be selected at random to commence the intervention (see Fig. 1). Centres will be randomly allocated to ‘step’, i.e. time of commencement of the intervention phase, by an independent statistician. This study design was chosen because the intervention involves the implementation of smoking cessation care as routine practice for all patients with cancer who smoke or have recently quit. Therefore, a simple randomised trial would not be feasible, and a cluster-randomised design was necessary. A stepped-wedge, cluster-randomised, controlled trial provides the same level of evidence as a standard, parallel, cluster-randomised controlled trial [30, 31] using fewer sites, while reducing the potential for contamination.

### Eligibility criteria

#### Study site and staff eligibility

Sites were recruited through professional networks. To be eligible, sites were required to have radiation oncology, medical oncology or relevant multidisciplinary clinics (e.g. head and neck). All medical, nursing, radiation therapy staff (and other relevant clinical staff tailored to the site) in the study cancer centres will be eligible to receive the implementation intervention and to participate in the study process measurement (i.e. staff surveys). Staff will deliver the smoking cessation care to patients as part of their normal role. Characteristics (e.g. demographics, disciplinary background and years of experience will be reported in the trial outcome paper).

#### Patient eligibility

##### Inclusion criteria

Patients eligible for inclusion will meet the following criteria:

(i) Aged at least 18 years
(ii) Diagnosed with any form of cancer in the prior 6 months (from date of pathology or radiology confirmation). Patients with metastatic disease are eligible, provided they have stable disease on current therapy and do not have an estimated prognosis of less than 12 months survival
(iii) Report combustible tobacco use either in the past 90 days, or in the 30 days prior to cancer diagnosis
(iv) Able to understand and speak English sufficiently to provide informed consent and participate in computer assisted telephone interviews; and have appropriate support (e.g. interpreter if required and available within their cancer service) to complete the study documentation
(v) Able to give informed consent
(vi) Accessing anti-cancer therapy from participating sites

##### Exclusion criteria

(i) Participants will be excluded if they are using e-cigarettes only (i.e. not smoking combustible tobacco products e.g. cigarettes) in the past 90 days, or in the 30 days prior to cancer diagnosis

### Recruitment and retention

Potentially eligible patients will be identified using clinic lists and at multidisciplinary team meetings by clinical staff and treating clinicians. Clinicians will be asked to refer potential participants to the study via the study research assistant, either in person or by providing contact details of people who have consented to be contacted by the research team. Additional recruitment strategies will include information flyers in clinics and sites will also mail study information packs to potentially eligible participants.

In addition, potential participants may self-refer by contacting the site research assistant. Potential participants will be provided with a participant information statement that includes the potential risks, their right to withdraw at any time and the details of data protection and confidentiality with sufficient time to ask questions. Patients will be recruited to the trial by site research assistants using informed consent (written or verbal). A signed (written or verbal) consent form will be obtained by site trial staff. Participants will be given the opportunity to agree or decline to being contacted for ancillary studies, without affecting participation in the main trial.

Computer-assisted telephone interviews (CATIs) will be used for the baseline and 7-month follow-up survey to aid in increasing retention rates. Monthly texts (to remind participants to inform the researchers if their contact details change) will be conducted to help maintain contact with participants.

### Participant timeline

This protocol is presented in accordance with the 2013 Standard Protocol Items: Recommendations for Interventional Trials (SPIRIT) Statement (see Supplementary Material). The schedule of enrolment, interventions, and assessments is summarized in Table 1.

**Table 1:** Stepwise procedures

Week	0	1	…	31	
Contact	*1*	*2*		*3*	
Enrolment					
Screening	X				
Informed consent	X				
Baseline assessment					
Computer-assisted telephone interview		X			
Follow-up assessment					
Computer-assisted telephone interview				X	
Biochemical verification of abstinence^a^ (breath carbon monoxide or salivary cotinine test)				X	
Post study period					
Medical record audit					X
Quitline data extraction^b^					X

^a^For reported abstainers

^b^For participants who report using the Quitline service

### Standard smoking care

During the baseline phase, each site will continue current care as usual. Standard care will differ at each site depending on their current usual practice. This may include screening some patients for smoking status at some time points and offering interventions such as referral to Quitline. Usual practice may also differ by clinicians within sites.

### Care to Quit intervention

#### ‘Ask, Advise, Act/Help’ (as per state-based recommendations)

**Fig. 2:**
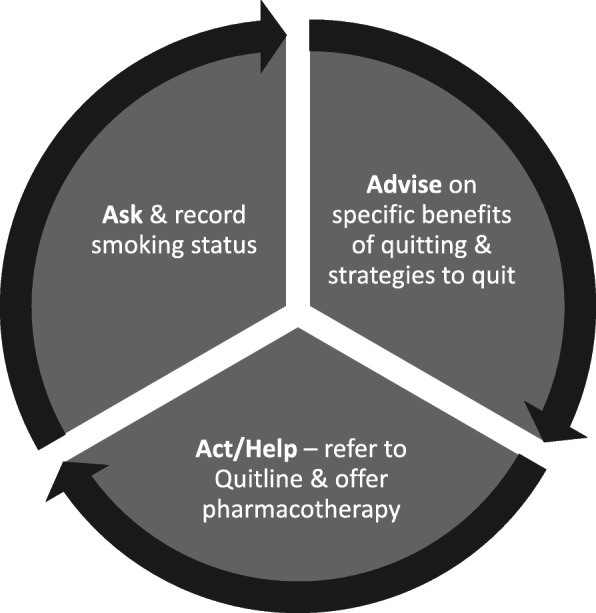
Cessation care model

All patients who smoke tobacco or have recently quit and who are receiving anti-cancer therapies at the study centres will be eligible to receive cessation care from clinic staff. The ‘Ask Advise Help’ model (see Fig. 2) is consistent with National Comprehensive Cancer Network recommendations [18] for evidence-based cessation support for cancer patients.

*Ask*: Assess and record smoking status (current smoker or recent quitter);

*Advise*: Give a personalised description of the specific benefits of smoking abstinence during and after treatment; endorse use of evidence-based support (telephone counselling and pharmacotherapy);

*Act*/*Help*: Offer referral to Quitline or other local cessation support service (including education around the benefits of Quitline); prescribe, provide or advise on pharmacotherapy options (nicotine replacement therapy (NRT) or varenicline) which are safe during treatment; and monitor progress. The above components of the cessation care model are the staff behaviours that our implementation intervention (described below) aims to target. A description of how these will be measured is provided in secondary outcomes (provision of smoking cessation care) and Table 2.

**Table 2:** Assessment schedule

	Baseline	7 months	
^a, b^Demographic and disease characteristics	X		
^a^Cancer site	X		
^a, b^Cancer treatment	X	X	
^a^Time since diagnosis	X		
^a^6 month prolonged abstinence (primary outcome)		X	
Saliva sample or CO breath test (those reporting abstinence)		X	
^a^Length of quit attempts		X	
^a^Heaviness of smoking index (including CPD)	X	X	
^a^Motivation to Quit	X	X	
^a^Self-efficacy to quit	X	X	
^a^Perceived benefit of quitting	X	X	
^a^Other smokers in household	X	X	
^a^Perceived social support	X		
^a^Risk perceptions	X	X	
^a^Use of pharmacotherapy	X	X	
Delivery of cessation support			
^a, b^Advice to quit	X	X	
^a, b, c^Proactive referral to Quitline	X	X	
^a, b^Prescription or provision of pharmacotherapy (including perceived acceptability)	X	X	
Mental health			
^a^PHQ-4 (+ 2 items from the PHQ to assess panic attack history)	X	X	
Substance use			
^a^Alcohol (AUDIT-C)	X	X	
^a^Cannabis use with tobacco q	X	X	
^a^Cannabis (First question of CUDIT)	X	X	
Quality of life			
^a^FACT–G7	X	X	
Financial effects			
^a^Financial stress scale	X	X	
^a^Financial distress thermometer	X	X	
Patient process measures			
^a^Patient recall of advice to quit	X	X	
^a^Patient recall of referral	X	X	
^a^Patient recall of pharmacotherapy prescription or provision	X	X	
^a^Attitudes and acceptability of receiving cessation support	X	X	
Cost data			
^g^Resource development, training/meetings, equipment/consumables		X	
	Baseline	Intervention (12 months duration)	Post-intervention (9–12 months duration depending on step)
Staff Process measures			
^d^Advice to quit	X	X	X
^d^Perceived competence with providing advice to quit	X	X	X
^d^Proactive referral to Quitline	X	X	X
^c^Number of calls and total minutes of completed calls	X	X	X
^a^Quality of contacts with Quitline and reasons for non-use	X	X	X
^d^Proportion of smokers with whom staff endorse Quitline	X	X	X
^c^Interstate comparability of Quitline protocols		X	
^d^Prescription or provision of pharmacotherapy	X	X	X
^d^Staff attitudes to pharmacotherapy	X	X	X
^d^Staff acceptability of providing cessation support	X	X	X
^d^Training in smoking cessation care	X	X	X
^d^Experience and perceptions of Care to Quit intervention		X	X
^e^Perceived role of system level factors (staffing, leadership, technology, infrastructure)	X	X	X
^f^Acceptability, experience of delivering smoking cessation counselling to participants			X

^a^CATI

^b^Medical record audit

^c^Quitline data extraction

^d^Online staff survey

^e^Key informant interviews

^f^Quitline counsellor interviews

^g^Project records

The model is relevant for current smokers and people who have recently quit smoking, including access to NRT as a relapse prevention strategy. As people with cancer are likely to report abstinence when in fact they are still smoking [22], it is important to ensure all recent quitters have evidence-based forms of cessation support ready at hand. The roles of oncologists, physicians, nurses, radiation therapists, hospital pharmacists and other relevant staff in the delivery of ‘Ask Advise Help’ will be tailored by the site staff to reflect the local context as part of the intervention process. Pre-existing skills, expertise and experience relevant to provision of smoking cessation care will differ across staff.

### Implementation of Care to Quit

#### Intervention phase: implementation (see Fig. 3 below)

The implementation intervention has been developed following the Theoretical Domains Framework (TDF) [29]. The TDF is a validated comprehensive framework for identifying barriers and enablers to behaviour change and includes 14 theoretical domains. We used this framework in preliminary work that included patient surveys, staff surveys, a pilot study and consensus processes [26], and prior unpublished work (Sherwood, Tzelepis, Day and Paul et al, unpublished). The strategies also address the need to adapt intervention content to the contexts being encountered [32]. Further detail is provided in a separate manuscript (Tait et al. unpublished) describing intervention development using the TDF and APEASE criteria (affordability, practicability, effectiveness and cost effectiveness, acceptability, side effects/safety and equity).

**Fig. 3:**
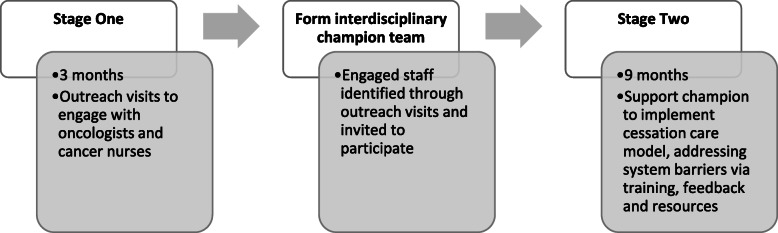
Implementation intervention stages

The overall duration of intervention implementation for the cessation care model is 12 months.

#### Stage 1 (3 months): building staff capability and motivation and identifying champions

Small group and individual outreach visits [33, 34] with oncologists, physicians and lead cancer nurses will be delivered by a behavioural scientist with smoking cessation expertise, and ideally a clinician, e.g. oncologist and a Quitline counsellor. At least one small group visit (30 min) and one individual visit per person identified by research team and sites as integral to implementation (e.g. head of department, senior staff member; 1 h) will be conducted across stage 1 (i.e. over 3 months).

The trainers’ specific backgrounds and expertise will be reported in the trial outcome paper. The visits will be interactive [35], use behavioural principles and incorporate the use of brief persuasive videos from discipline-relevant opinion leaders.

The visits will address (i) the evidence of cessation benefits specific to the types of patients the oncologist sees; (ii) evidence for multi-session specialist telephone support (i.e. Quitline) including how counselling is now tailored to support people with cancer; (iii) evidence regarding cessation pharmacotherapy during cancer treatment; (iv) suggested scripts and modelling of brief interactions for succinctly discussing cessation benefits and endorsing Quitline; (v) sensitive timing and framing of stop smoking messages; and (vi) reviewing recent consultations and managing challenging cases.

#### Stage 2 (9 months): identifying and implementing cessation care models/pathways

During stage 1, an inter-disciplinary team interested in championing smoking cessation care will be identified and invited to form an interprofessional, multi-disciplinary team of project champions at each cancer centre. The team will be supported by regular teleconferences with the researchers to identify how to implement ‘Ask Advise Help’ within existing patient pathways. Researchers will work collaboratively with sites to develop models within each site.

Sites will be supported (for the duration of stage 2) with a suite of evidence-based implementation strategies tailored to the needs of the individual site. Implementation strategies include (i) feedback [36] of current rates of care using data collected in the baseline phase, (ii) evidence-informed training for staff not involved in stage 1 (delivered via multiple formats such as face to face and existing online Quit and health departmental training, with refreshers and updates at regular intervals) and (iii) educational tools [23] for use with patients. The method of providing pharmacotherapy will vary as not all centres are able to provide this directly to outpatients. Where the medication cannot be supplied directly to the patient (e.g. using a hospital pharmacist-led or nurse-led approach), a prescription can be written by the treating doctor. Referral to the patient’s GP for prescription of pharmacotherapy may also occur. Ongoing support will be provided to sites to support adherence to the collaborative and tailored models developed.

Staff will be invited to complete surveys online (or print if preferred by the site) at each study phase (baseline, intervention, post-intervention) to collect process measures data, experiences with and perceptions of the Care to Quit intervention and provision of smoking cessation care. Due to the stepped-wedge design of the trial, sites will enter intervention and follow-up periods at varying timepoints. Sites 1–3 will enter intervention at 6 months post baseline, sites 4–6 at 13 months post baseline and sites 7–9 at 19 months post baseline (Fig. 1). Staff surveys (Table 2) will therefore occur at varying timepoints from baseline across sites. The intervention period questionnaires will be completed mid-intervention period (i.e. 6 months post beginning of intervention period) for each cluster of sites (i.e. 13 months post baseline for sites 1–3, 19 months post baseline for sites 4–6 and 25 months post baseline for sites 7–9). The post-intervention period questionnaires will be completed at the beginning of the post-intervention period (19 months post baseline for sites 1–3, 25 months post baseline for sites 4–6 and 28 months post baseline for sites 7–9).

As the support provided at stages 1 and 2 will be delivered following assessment of site need (including current procedures, existing staff skills and experience) and tailored accordingly, further details of intervention dose (i.e. consultations, meetings, training materials provided) and mode of delivery (e.g., individual vs group, powerpoint presentations, videos) will be provided in the trial outcome paper. Project records will be used to document staff attendance at meetings and training. The delivery of smoking cessation support to patients will be implemented as part of routine care and tailored to site. Therefore, this may occur individually face to face at outpatient clinics, via telephone and/or telehealth and the amount or ‘dose’ of the support will differ.

### Primary outcomes

As recommended by the Society for Research on Nicotine and Tobacco expert workgroup [37], the primary outcome is defined as self-reported 6-month prolonged abstinence (with no relapse, i.e. 7+ days of prolonged smoking) at the 7-month follow-up, allowing participants up to 1 month to stop smoking.

### Secondary outcomes

Secondary outcomes assessed at 7-month follow-up will include:

#### Smoking

- Biochemically verified abstinence from smoking (for participants reporting prolonged abstinence at the 7-month follow-up, and no smoking in the last week). Measured via breath carbon monoxide monitor or salivary cotinine test.
- Length of quit attempts (defined as stopping smoking for a period of 24 h or more). Past quit attempts are predictive of future attempts [38].
- Nicotine dependence: assessed using the two item Heaviness of Smoking Index (HSI [39]). It uses a six-point scale calculated from the number of cigarettes smoked per day (1–10, 11–20, 21–30, 31+) and the time to first cigarette after waking (< 5, 6–30, 31–60 and 61+ min). Nicotine dependence is then categorized into a three-category variable: low (0–1), medium (2–4) and high (5–6).
- Daily consumption: cigarettes per day (CPD) as measured by item 1 of the HSI; ‘How many cigarettes do you typically smoke per day?’ HSI and CPD are strong predictors of quitting behaviour [40].

#### Mental health and quality of life

The Functional Assessment of Cancer Therapy-General rapid version (FACT-G7 [41]) is a 7-item measure for evaluating symptom/concern burden and quality of life (QOL) in advanced cancer patients over time. Despite beliefs that smoking cessation worsens mental health symptoms, previous studies indicate smoking cessation leads to no worsening and possibly improvement in mental health and quality of life; however, more research is required [42]. Research on QOL and smoking cessation amongst patients with cancer is very limited.

#### Provision of smoking cessation care

The outcomes described below will serve as a measure of staff adherence to the smoking cessation care model; Ask, Advise, Act/Help.

- Advice to quit: as measured by any indication of a personalised description of the specific benefits of smoking abstinence during and after cancer treatment or endorsed use of evidence-based support (telephone counselling and pharmacotherapy), extracted by medical record audit and patient self-report. Advice to quit increases the likelihood that an individual will make a successful quit attempt [43, 44] and is an indicator of whether the implementation intervention has changed clinicians’ practice.
- Proactive referral to Quitline (including education around the benefits of Quitline): as assessed by review of Quitline records, medical record audit and patient self-report. Receipt of telephone cessation support (Quitline) increases the likelihood that an individual will make a successful quit attempt [45] and is an indicator of whether the implementation intervention has changed clinic practice.
- Prescription or provision of pharmacotherapy (including NRT and stop smoking medications) as assessed by medical record audit and patient self-report. NRT and stop smoking medications have a strong evidence base to assist smoking cessation [46–48] and provision of these is an indicator of whether the implementation intervention has changed clinic practice.

#### Cost effectiveness

Cost effectiveness will be measured by cost per successful quit attempt from a service-provider perspective. These data will be sourced from project and site records.

### Process measures

- Advice to quit: as assessed by staff self-reported delivery of advice, patient self-report (including the source [doctor, nurse, radiation therapist, other], and content [whether cancer-related reasons for quitting were provided] and impact of the advice).
- Perceived competence with providing advice to quit: as measured by staff self-report.
- Referral to Quitline and quality of contacts: as assessed by staff and participant self-report.
- Provision or prescription of pharmacotherapy and attitudes: as assessed by patient and staff self-report.
- Attitudes and acceptability of providing/receiving cessation support: as measured by staff and patient self-report.
- System perspectives: including staffing, workload, leadership, technology, infrastructure, as assessed by interviews with key informants.
- Training in providing smoking cessation care: staff will be asked about if they have received smoking cessation care training and the form of training received.
- Experience and perceptions of Care to Quit intervention: as measured by staff self-report.

In addition to being important intermediate/process indicators, these data will be used to fully describe the implementation process as recommended for implementation studies [49] and measure differences in resource utilisation between the intervention and control groups.

### Covariates

- Patient-reported sociodemographic characteristics (e.g. gender and age).
- Motivation to quit: the motivation to stop scale [50] has been adapted to assess motivation to quit amongst participants currently smoking at baseline and/or follow-up. The number of response options have been lessened from the original scale to: ‘I don’t want to stop smoking’, ‘I think I should stop smoking but don’t really want to’, ‘I want to stop smoking but I don’t know when I will’, ‘I really want to stop smoking and intend to in the next month’, ‘I really want to stop smoking and intend to in the next 6 months’ and ‘None of the above’. Participants who have stopped smoking at baseline or follow-up will be asked ‘How motivated are you to stay quit long-term and become permanently smoke-free?’ Response options range from ‘Not at all motivated’ to ‘Extremely motivated’.
- Quitting self-efficacy: participants currently smoking at baseline or follow-up will be asked the self-efficacy item from the International Tobacco Control four country survey [51] ‘If you decided to give up smoking completely in the next 6 months, how sure are you that you would succeed?’, which has been adapted from a 5- to 4-point scale of response options. Participants who have stopped smoking at baseline and/or follow-up will be asked ‘How confident are you that you will be able to stay quit long-term and become permanently smoke-free?’ Response options range from ‘Not at all confident’ to ‘Extremely confident’.
- Perceived benefits of quitting: participants will be asked at baseline and 7-month follow-up ‘How much do you think it would benefit your health if you were to quit smoking now or stay smoke-free?’ Response options range from ‘Not at all beneficial’ to ‘Extremely beneficial’.
- Smoking status of household members: at baseline and 7-month follow-up, participants will be asked ‘Besides yourself, do any other members of your household smoke cigarettes or use other tobacco products?’
- Perceived social support: at baseline, participants who are currently smoking/recent quitters will be asked ‘How much do you feel you could count on your family and friends to support you if you tried to quit smoking/stay smoke-free in the next 6 months?’ Response options include ‘Not at all’, ‘Somewhat’ and ‘A great deal’.
- Risk perceptions: at baseline and 7-month follow-up, participants will be asked to rate on a 5-point scale (ranging from ‘Strongly agree’ to ‘Strongly disagree’) how much they agree with the following statements: ‘Quitting smoking after a cancer diagnosis can make cancer treatments more effective’; ‘Quitting smoking after a cancer diagnosis increases the risk of side effects from cancer treatment’; and ‘Quitting smoking after a cancer diagnosis reduces the risk of secondary cancer or cancer recurrence’.
- Use of pharmacotherapy: at baseline and 7-month follow-up, participants will be asked ‘Have you used any of the following medications to help you quit/stay smoke-free?’ Response options will include ‘nicotine replacement products (e.g. gum, patches, lozenges, mouth spray, inhalator), Champix (i.e. Varenicline), Zyban (i.e. Bupropion)’.
- Financial distress: at baseline and 7-month follow-up, participants will complete the Financial Stress Scale [52, 53]. This scale asks participants ‘At any time since your most recent primary cancer diagnosis has any of the following happened to you because of a shortage of money?’ Response options include, for example: ‘Could not pay the mortgage or rent on time’. Based on prior research, this scale has been adapted to remove the ‘unable to heat/cool the home’ response option [54] and includes additional financial stressors related to a cancer diagnosis (e.g. ‘Could not pay for medical consultations or tests with GP or specialist’) [55]. Participants will also complete a financial distress thermometer, which asks ‘On a scale of 0 to 10, with 10 being extreme distress and 0 being no distress, please pick a number that describes how much financial distress you have been experiencing in the past week including today’.
- Mental health: as measured by the Patient Health Questionnaire-4 (PHQ-4 [56]) and an additional 2 items from the PHQ to assess panic attack history (‘In the last four weeks, have you had an anxiety attack—suddenly feeling fear or panic? Has this ever happened before?’). The PHQ-4 is a valid ultra-brief tool for detecting both anxiety and depressive disorders.
- Substance use: The Alcohol Use Disorders Identification Test—Brief (AUDIT-C [57]) is a 3-item screening tool used to identify hazardous alcohol use or active alcohol use disorders. In addition, participants are asked ‘Have you used cannabis for medicinal and/or recreational purposes? (over the last 6 months)’ with response options ‘Yes, No’, ‘How often did you use cannabis?’ with response options ‘Monthly or less, 2–4 times a month, 2–3 times a week, 4 or more times a week’ and ‘Do you mix tobacco with your cannabis?’ with response options ‘Yes, always or nearly always,’ ‘Yes, sometimes’ or ‘No, never or very rarely.’

#### Patient-completed measures

CATIs will occur within 2 weeks (baseline) and 7 months of recruitment.

#### Staff-completed measures

Online (or print if preferred by site) surveys will occur at the beginning of each study phase (baseline, intervention, post-intervention). These will be self-completed.

The assessment schedule is presented in Table 2.

### Sample size

The trial will recruit 2160 smokers (40 per site per 6 months over 3 years for each of nine sites). This sample size will provide approximately 80% power to detect a difference of 7% in smoking cessation (i.e. during the baseline phase 7% of those who were smokers or recent quitters at recruitment will achieve 6 months prolonged abstinence, while during the intervention phase 14% of those who are still smoking at diagnosis will achieve 6 months abstinence) at an alpha level of 0.05, assuming an intra-cluster correlation of 0.01 and a worst-case scenario of cluster autocorrelation equal to zero. This sample also provides over 90% power to detect each of the differences previously stated for the secondary outcomes.

### Randomisation

At each intervention ‘step’, 3 sites will be selected at random to commence the intervention. Centres will be randomly allocated to ‘step’, i.e. time of commencement of the intervention phase, by an independent statistician. For pragmatic reasons, three sites will be treated as their own cluster due to the concern for contamination if sites participated in the intervention at different times.

### Blinding

Participants, interviewers conducting the CATIs and data analysts will be blinded to the intervention phase. Because of the nature of the intervention, it is not possible to blind clinicians at participating sites or trial investigators to the sites’ allocation to ‘step’, i.e. time of commencement of the intervention phase once randomised. Participants will be blinded to treatment allocation.

### Data management

All data will be entered electronically via e-case report form using Research Electronic Data Capture (REDCap) tools [58] hosted at Hunter New England Local Health District NSW on a secure server. REDcap is a secure, web-based application designed to support data capture for research studies. The lead investigator (and/or delegate) and trial coordinator will conduct ongoing data checking and cleaning. Participant personal details will be accessed, used, and stored according to relevant legislations. Access to external health data (e.g. Quitline, health records) will only occur with the consent of the participant in accordance with protocols of relevant external agencies.

The current study is a trial of a non-invasive clinician-delivered smoking cessation support intervention. Clinicians will be trained in support strategies. These strategies will be delivered within the context of standard patient consultations. It does not involve administration of medicine or experimental therapeutic devices. In light of this an independent data safety monitoring board will not be convened.

### Evaluation

#### Statistical methods

Independent and blinded statisticians from the CReDITSS Unit at the Hunter Medical Research Institute, Australia, supervised by CI Barker, will conduct analyses of the primary and secondary outcomes.

Logistic regression within a generalised linear mixed models framework will be used to model the primary and secondary outcome measures. All models will adjust for time using a fixed effect that describes the phase of the study (pre-intervention and each of the 3 steps). Variation between sites will be accounted for using a random effect for site and repeated measures on sites will be accounted for using a random effect for time within site. To estimate the intervention effect, a variable indicating when the intervention was active for a given site and time will be included in the models. As the timing of the 7 month CATI for some participants overlaps with intervention commencement, the analyses of 6 months abstinence will include a 6-month offset to the coding of the intervention indicator variable to allow the full effect of the intervention to be in place before estimating its effect.

There are no plans for interim analyses.

##### Cost-effectiveness evaluation

Using a health services perspective, incremental cost-effectiveness ratios will be calculated from the cost per person quit in the intervention phase (i.e. self-reported 6-month abstinence from smoking) compared to usual care in the pre-intervention phase. Costs for the delivery of the intervention (e.g. clinician training, smoking cessation resources, NRT/pharmacotherapy where appropriate) will be derived directly from trial data and added to the costs of clinician time (e.g. oncology nurses) in usual care. Exploratory, modelled analyses will examine the potential impacts of smoking cessation (intervention versus usual care) on the cost per quality adjusted life year saved, using mortality relative risk estimates derived from published studies of survival by post-diagnosis smoking cessation (however these studies are likely subject to confounding by indication for quitting and as such modelled results based on these data will be interpreted cautiously). Modelled analyses will include estimates of the health-system costs of cancer [59] and health state utilities for cancer survivors [60]. Sensitivity analyses will address potential variation in costs and the survival benefits due to smoking cessation.

### Qualitative evaluation

A nested qualitative study will be conducted. All interviews (key informants and Quitline counsellors) will be audio recorded, transcribed and a general inductive approach will be taken for the analysis [61]. Interviews will be guided by semi-structured interview guides.

#### Key informant interviews

Semi-structured interviews will be conducted with *n* = 45 staff participants (*n* = 5 per site). Potential participants will be purposively selected at each timepoint (baseline, intervention, post-intervention) to be invited for interview. Potential participants will be identified by the site principal investigator in consultation with the research team and advised by the principal investigator that they will be contacted by the research team with an invitation to participate in an interview. Eligible participants will include cancer services directors, heads of departments, directors of cancer nursing and team leaders. Interviews will explore the perceived role of system-level factors (e.g. staffing, leadership, technology, infrastructure) which can affect implementation. The interviews will also evaluate the method of multiple outreach visits in identifying and motivating an interdisciplinary group of champions. Participants will have the option of reviewing the interview transcript and editing their responses prior to analysis.

#### Quitline counsellor interviews

Interviews will evaluate the experience of delivering counselling to study participants amongst Quitline staff. It is anticipated that semi-structured individual and/or group in-depth interviews will be conducted with Quitline counsellors to enable data collection to reach saturation and for key themes to be identified [62]. Interviews will explore their experience of delivering counselling and its strengths and weaknesses from their perspective. Interviews will be conducted face to face, via telephone or videoconference depending on practicality and participant preference. Participants will have the option of reviewing the interview transcript and editing their responses prior to analysis.

Recruitment will be undertaken via email from Quitline managers to all eligible Quitline staff (those that delivered counselling to Care to Quit participants) asking them to participate in an interview. Their participation will be optional.

### Harms

Adverse events will be monitored by study sites (hospitals) as per their usual protocols.

### Protocol amendments

Each study site will only be able to start data collection once the relevant Ethics Committee and research governance approval is obtained. In the case of proposed protocol changes, an amendment will be submitted to the Ethics Committees for approval, and the trial coordinating centre will ensure all study staff are provided with new documentation. Any significant protocol changes will be updated on the ANZCTR site and reported in the final outcomes paper.

### Confidentiality

The trial will be conducted in accordance with applicable Privacy Acts and Regulations. Data that identify any trial participant will not be revealed to anyone not directly involved in the trial or the clinical care of that participant. An exception is where the trial participant has provided written consent for his/her records to be included in source document verification.

### Access to data

All data will be considered the property of the trial chief investigator who, in consultation with the trial management committee, will be responsible for presentations and publications arising from this trial.

### Dissemination policy

Trial findings will be summarized and provided to participants who have indicated they would like a copy of the results. The trial management committee will be responsible for decisions regarding presentations and publications arising from this trial according to agreed Authorship, Publication and Spokesmanship Guidelines.

Access to data during the trial will be limited to the trial management committee and appropriate regulatory bodies.

## Discussion

Given the risks of greater morbidity, tumour recurrence and mortality in the 25% or more of people with cancer who continue to smoke [13, 27], the untapped benefit of achieving smoking cessation amongst people with cancer is substantial. Oncology clinicians and health professionals have a pivotal role to play in delivering best practice smoking cessation care. In addition to the likelihood of successfully improving the implementation of smoking cessation care for people with cancer, the Care to Quit trial will advance the field of implementation science in three ways. Firstly, the initial intervention stage addresses the capability and motivation of staff intensively via outreach visits before study teams are formed. Secondly, outreach visits (personal visits by a trained person to professionals in their own settings) are much less studied than strategies such as feedback. While outreach visits have been found effective for changing prescribing [63], it is not clear whether this approach is effective for changing other aspects of behaviour (e.g. content and nature of consultation advice). Third, champions are assumed as essential but there is little data on how to identify or develop leaders for practice change [64–66]. The cost effectiveness evaluation will provide valuable information for state and national governments that commission or fund health care regarding the costs of upscaling the implementation (should it be effective).

### Limitations

Site clinicians will not be blind to participant allocation, which may introduce bias into their documentation of the provision of smoking cessation care into patient medical records or their own self-report in surveys. However, record audits have been found to be a valid measure of care provision [67] and these will also be supplemented with patient recall of receipt of smoking cessation care.

It would be useful in future studies to follow the participants over a longer timeframe to measure longer-term health and other benefits.

## Supplementary Information

**Additional file 1.**

## Data Availability

Not applicable.

## Abbreviations

CATI: Computer-assisted telephone interviews
NRT: Nicotine replacement therapy
QOL: Quality of life
REDCap: Research Electronic Data Capture
SPIRIT: Standard Protocol Items: Recommendations for Interventional Trials
